# 

**Published:** 1921-09-24

**Authors:** 


September 24 1921] [Price Twopence

### The Hospital

The Workers' Newspaper of Administrative Medicine and Institutional Life, Administration, National Insurance and Health.

CONTENTS

**From Weekly Journal to Monthly Review...** ... 421

**The Limitations of Dentistry...** ... ... ... 422

**Purifying Tidal Rivers ...** ... ... ... ... 422

**Notes of the Week** ... ... ... ... .. 423
A Self-Penalised Institution—Lady Askwith and the Health Ministry—Our Water Supply—An Appeal—The Coming Winter—Walsall General Hospital—The Effect of Closing Beds—A Novel Campaign—A Useful Work Completed—Endowed Beds and Hospital Visitors.

**The Rockefeller Foundation ...** ... ... ... 425

Past and Future Work.

**An Eighteenth-Century Hospital** ... ... ... 425

**The Energy Values of Food ..** ... ... ... 426

**A Bundle of Old Reports** ... ... ... ... 427
Hospital Conditions in London Sixty Years Ago.

**Eye Injuries in Film Production** ... ... ... 428

**Health and Education in Yugo-Slavia** ... ... 429

**The Institutional Library** ... ... ... ... 429
Electro-Therapeutics for Practitioners—Gynæcology—A Text-Book of Special Pathology—String Figures: A New Amusement for Everybody—Pope's Manual of Nursing Procedure.

**The Editor's Letter-Box** ... .. ... ... 430
Scientific Massage *versus* Ineffectual Rubbing—Sister-Tutors for Mental Hospitals.

**The Matrons' and Sisters' Department** ... ... 431

Grants for Nurse-Training Schools.

The Amended Syllabus of Nursing Education.

**Round the Hospitals** ... ... ... ... ... 432

**Letters to a Nurse** ... ... ... ... ... 433

II.—The Panel Doctor.

**The College of Nursing (Limited by Guarantee)** 434

**Matrons' and Sisters' Appointments** ... ... 434

**St. Marylebone General Dispensary** ... ... 434

**The Plight of a Bombay Hospital ...** ... ... 434

